# The Controversial Role of IL-33 in Lung Cancer

**DOI:** 10.3389/fimmu.2022.897356

**Published:** 2022-05-12

**Authors:** Keshan Yang, Cheng Tian, Chengliang Zhang, Ming Xiang

**Affiliations:** ^1^ Department of Pharmacology, School of Pharmacy, Tongji Medical College, Huazhong University of Science and Technology, Wuhan, China; ^2^ Department of Pharmacy of Tongji Hospital, Tongji Medical College, Huazhong Science and Technology University, Wuhan, China

**Keywords:** IL-33, immune regulation, pulmonary fibrosis, lung cancer, dual regulation

## Abstract

Interleukin-33 (IL-33) belongs to the interleukin-1 (IL-1) family, and its structure is similar to IL-18. When cells are damaged or undergo necrosis, mature form of IL-33 is secreted as a cytokine, which can activate the immune system and provide danger signals. The IL-33/ST2 signaling pathway is composed of IL-33, suppression of tumorigenicity 2 (ST2), and IL-1 receptor accessory protein (IL-1RAcP). IL-33 has been reported to be strongly associated with lung cancer progression, and can exhibit opposite effects on lung cancer under different conditions. In this review, we have summarized the structure and basic functions of IL-33, its possible function in immune regulation, and its role in pulmonary fibrosis as well as in lung cancer. We have highlighted the dual regulation of IL-33 in lung cancer and proposed potential lung cancer treatment regimens, especially new immunotherapies, based on its mechanism of action.

## Introduction

IL-33 plays an important role in type 2 innate immune responses and allergic inflammation by activating various target cells. It also plays a wide variety of roles in parasitic and viral host defense, tissue repair and homeostasis (1). A number of previous studies have indicated that IL-33 exerts anti-lung cancer effects under certain conditions, while driving lung cancer progression in other circumstances.

Lung cancer is a kind of carcinoma associated with high morbidity and mortality, and its morbidity is preceded only by breast cancer. According to the 2021 Cancer Statistics Report, there were 2.35 million new cases of lung cancer and about 1.31 million people died from lung cancer worldwide, in 2021 (2). Lung cancer, which contributes to approximately nearly 20% of all cancer deaths, is the main cause of cancer related mortality. The immunotherapy has been considered as promising therapy for lung cancer treatment, including immune checkpoint inhibitors such as Nivolumab and Durvalumab, which have been approved for the treatment of lung cancer (3). Additionally, cytokines such as IFN-α2a, IFN-α2b as well as IL-2 have been approved for cancer treatment (4).

IL-33 is able to promote the function of peripheral blood mononuclear cells (PBMCs), dendritic cells (DCs), CD8^+^ T cells and natural killer (NK) cells, which can in turn inhibit the tumor growth and metastasis (5). According to the existing research results, we believe that there is a possibility of clinically using IL-33 to specifically enhance the function of immune cells in the treatment of lung cancer.

## An Overview of Lung Cancer

### Lung Cancer Pathogenesis and Therapy

Lung cancer, like most other cancers, primarily arise due to the imbalance of cell proliferation and apoptosis caused by gene mutations, mainly due to the increased expression of C-myc, N-myc, and L-myc belonging to the myc gene family (6–8). There are two main subtypes of lung cancer, with small cell lung cancer (SCLC) accounting for 15% and non-small cell lung cancer (NSCLC) accounting for 85% of all the diagnosed cases (9). It has been reported that deletions or mutations of genes such as p53 and RB1 were mainly observed in SCLC (10, 11). As for NSCLC, mutations in epidermal growth factor receptor (EGFR), kirsten rat sarcoma viral oncogene (KRAS), mesenchymal-epithelial transition factor (MET) and other genes have been mainly observed (12).

There are several main treatment options for NSCLC. Lobectomy, which removes a single lobe, is considered to be the optimal procedure for early-stage NSCLC. And the survival is generally higher with lobectomy than with limited resection (13, 14). Administration of chemotherapy before surgery, which is also known as neoadjuvant chemotherapy, has the potential of inhibiting micrometastases, downstaging of the tumor as well as improving tolerability compared with the adjuvant approach. According to existing results, neoadjuvant chemotherapy is feasible and immunotherapies may be an optimal treatment strategy for early‐stage NSCLC in the neoadjuvant setting (15). On the contrary, adjuvant chemotherapy that administrates chemotherapy after surgery has been reported to help prolong the survival time of patients receiving surgical resection. Targeted therapy, which includes EGFR inhibitors or antibodies as well as vascular endothelial growth factor receptor (VEGFR) inhibitors, is another option for NSCLC patients (16). Immunotherapy has dramatically changed the landscape of treatment of NSCLC. To limit damage to healthy cells, the process of the immune system recognizing and destroying neoplastic cells is highly regulated by an equilibrium of activating and inhibitory pathways (17). One of these pathways is the programmed death 1 (PD-1)/programmed death ligand 1 (PD-L1) axis. The PD-1 is a transmembrane receptor that is expressed in a variety of tissues. Its ligand, PD-L1, is expressed in T cells, and this interaction leads to the inactivation of T cells. Malignancies can overexpress PD-L1 as a mechanism of defense against the host’s immune system (16). Immune checkpoint inhibitors (ICIs) such as PD-1 or PD-L1 blockades have been approved for the treatment of NSCLC, FDA-approved immunotherapeutic drugs and their targets are listed in (**Table 1**) (3).

**Table 1:** PD-1 inhibitor Nivolumab (or Opdivo) have been approved for treatment of NSCLC and SCLC, another PD-1 inhibitor Pembrolizumab (or Keytruda) have been approved for SCLC treatment.

Drug	Target	Disease
Nivolumab	PD-1	NSCLC & SCLC
Opdivo (Nivolumab)		NSCLC & SCLC
Pembrolizumab		NSCLC
Keytruda (Pembrolizumab)		NSCLC
Atezolizumab	PD-L1	NSCLC & SCLC
Tecentriq (Atezolizumab)		NSCLC & SCLC
Durvalumab		NSCLC & SCLC
Imfinzi (Durvalumab)		NSCLC & SCLC

PD-L1 inhibitors Atezolizumab (or Tecentriq) and Durvalumab (or Imfinzi) have been approved for treatment of NSCLC and SCLC.

As for SCLC, no major advances in its treatment have occurred over the past 30 years (18). The first‐line treatment in SCLC recommended by the United States and Europe is 4‐6 cycles of etoposide plus cisplatin or carboplatin and the FDA has approved the PD‐L1 inhibitor atezolizumab in combination with carboplatin and etoposide as a first‐line therapy for SCLC (19). Concurrent chemoradiotherapy is the standard of care in limited‐stage disease (LD) SCLC, but the optimal radiotherapy schedule and dose remains unclear (19). Surgery is an option for LD SCLC patients that have gone through mediastinal sample collection and diagnostic computed tomography analysis (20). Prophylactic cranial irradiation could reduce the incidence of brain metastasis in both limited‐ and extensive‐stage patients, but the patients would have lower quality of life due to the toxicity of the therapy (21–23). Palliative care on extensive-stage disease (ED) SCLC is expected to prolong the survival time, improve quality of life, as well as minimize the risk of symptoms associated with disease.

### The Effects of Various Lung Cancer Treatment Modalities

Surgery is a standard option for early-stage NSCLC patients. The pathologic complete response rate in a survey of NSCLC patients undergoing surgery was reported to be 60% (24). As for radiotherapy, a clinical survey of patients with stage I NSCLC who received stereotactic radiotherapy (SRT), the median overall survival was 2.8 years, and the 1- and 2-year survival rates were observed to be 81% and 64%, respectively (25). In a survey of NSCLC patients receiving chemotherapy, the objective response rate was 32% (26), which was much lower compared with other treatments. As chemotherapy alone is not as effective as other treatments, it is usually combined with other therapies for lung cancer. Unlike chemotherapy, targeted therapy significantly helps improve patient survival. For instance, the median progression-free survival (PFS) of patients with stage IIIB/IV lung adenocarcinoma treated with afatinib was 13.6 months (27). Additionally, a meta-analysis summarized 13 phase 3 trials in which an EGFR TKI was compared with platinum-based chemotherapy, PFS was significantly prolonged, whereas no effect on overall survival was observed (28). Immunotherapy combined with chemotherapy showed impressive potential in lung cancer treatment. A study which analyzed the efficacy of Pembrolizumab (Pembro) and Atezolizumab (Atezo) combined with carboplatin and nab-paclitaxel (CnP) as first-line treatment of advanced squamous NSCLC indicated that the overall survival and progression-free survival of treatment Pembro + CP/CnP reached 15.9 months and 6.4 months, and Atezo + CnP reached 14.0 months and 6.3 months (29). These findings show that immunotherapy is quite effective, so it makes sense to seek new immunotherapy for lung cancer treatment.

## An Overview of IL-33

### Structure and Basic Function of IL-33

IL-33 protein consists of three distinct functional domains; nuclear domain, central domain and IL-1-like cytokine domain (30). Such a structure makes IL-33 a dual-type protein, which can act both as a pro-inflammatory cytokine and a nuclear factor capable of regulating transcription. Under basal conditions, the chromatin-binding motifs can localize IL-33 protein into the nucleus by interacting with histone H2A-H2B dimers and bind IL-33 to chromatin (31). The central domain of IL-33 contains protease cleavage sites that are sensitive to neutrophil-derived and mast-cell-derived proteases (32, 33). The IL-1-like cytokine domain of IL-33 binds to ST2 on target cells and mediates cytokine activity (34, 35).

In human lung, IL-33 is constitutively and abundantly expressed in endothelial cells, epithelial cells, fibroblasts, myofibroblasts, mesenchymal as well as smooth muscle cells (1). Epithelial progenitor cells were reported to express IL-33 during chronic obstructive pulmonary disease (36). And in lung inflammation such as asthma, IL-33 expression increased in epithelial cells and airway smooth muscle cells (37, 38). Serum level of IL-33 in lung cancer patients was reported similar, higher or lower compared with that in healthy volunteers in different studies (39–41), reflecting that the role of IL-33 in lung cancer is complex.

As a nuclear factor, IL-33 can effectively bind to the chromatin to inhibit the various inflammatory responses. As a cytokine, given the right combination of signals and exposure to the cellular damage, stored IL-33 is released to interact with its receptor ST2, thus triggering danger-associated responses and act as a cellular “alarmin” (42). Constitutive expression of preformed IL-33 in the endothelium and epithelial barriers allows it to respond rapidly to damage caused by viruses or parasites, allergens or intrinsic injury (1). The full-length IL-33_1-270_ in the nucleus can thereafter inhibit the inflammatory response after interacting with histones, but when the cell is damaged or necrotic, it is sheared to generate a series of active fragments that can be released outside the cell to promote inflammatory responses, activate the immune system, and thereby provide damage signal (32, 33, 43). Although the mechanism remains unclear, it has been reported that ATP, uric acid, oxidative stress as well as protease-activated receptor protein activation lead to IL-33 secretion (44–46), which involves an increase in intracellular calcium and/or P2 purinergic receptor (47).

IL-33/ST2 signaling causes phosphorylation of GATA3, which recruits more GATA3 and RNA polymerase II to the Foxp3 promoter. GATA3 binds to the ST2 promoter, enhancing ST2 on the surface Tregs, thus promoting Treg function through enhancing TGF-β1-mediated differentiation (48). A study reported that peroxisome proliferator-activated receptor gamma (PPARγ) is involved in the interaction between IL-33 and group 2 innate lymphoid cells (ILC2) (49). By activating ornithine decarboxylase, which catalyses the synthesis of polyamines, IL-33 induces M2-like macrophage polarization (50). IL-33 is also able to induce the gene expression of Th2-associated cytokines such as IL-5 and IL-13 (34).

### IL-33 Receptor, the Mechanism of IL-33/ST2 Pathway and its Relationship With Tumors

Suppression of tumorigenicity-2 (ST2) is divided into four different subtypes: sST2, ST2L, ST2V and ST2LV. Among them, sST2 possesses no transmembrane sequence and can be secreted outside the cell. It is a soluble ST2 and is only expressed in mast cells and fibroblasts. ST2L has a transmembrane sequence and cannot be secreted outside the cell. It is a transmembrane ST2 and is primarily expressed in fibroblasts, mast cells, eosinophils, DCs, NK cells, NKT cells, helper T cell 2 (Th2) lymphocytes, and activated macrophages (51). ST2V is characterized by the absence of an immunoglobulin-like motif and alternative splicing of the C-terminal portion of ST2 inserting a new exon (52). ST2LV is a new secreted soluble and N-glycosylated variant of the ST2 gene product (53). ST2 primarily exists as a splice variant in two distinct forms: sST2 acts as a decoy receptor to sequester free IL-33 and blocks IL-33 signal, whereas ST2L can activate the MyD88/NF-κB signaling pathway, consequently enhancing the function of Th2 lymphocytes, Tregs, mast cells and ILC2s (54).

The mechanism of action of IL-33/ST2 pathway can be summarized as follows (55): (A) The action of IL-33 signaling. IL-33 binds to ST2L and leads to the recruitment of IL1-RAcP (56). ST2L interact with IL1-RAcP to facilitate the recruitment of MyD88, IRAK1, IRAK4, and TRAF6. These molecules activate NF-κB, JNK, ERK, and p38, leading to the expression of different gene-encoding cytokines, chemokines, and growth factors. (B). The attenuation of IL-33 signaling. Single-immunoglobulin interleukin-1 receptor-related molecule (SIGIRR) is able to disrupt the ST2L/IL1RAcP heterodimer. Extracellular IL-33 can be sequestered by sST2 acting as a molecular decoy.

On one hand, binding of IL-33 to ST2L can promote the tumor cell growth, proliferation and metastasis by inducing Th2-type cytokines and promoting the accumulation of immunosuppressive cells in tumors (57). It is reported that Rab37 small G protein regulates exocytosis of sST2, resulting in the shift of macrophages phenotype from M2-like to M1-like, and thereby inhibiting tumor growth (58). On the other hand, it leads to an inflammatory gene transcription and ultimately to the production of inflammatory cytokines/chemokines and mounting of an adequate immune response (59). IL-33 signaling enhances CD8^+^ T cell activity response to inhibit the tumor growth (60). It is also reported that IL-33 in combination with IL-12 promoted IFN-γ production by both invariant natural killer T (iNKT) cell and NK cell, thus enhancing innate cellular immune responses (61). However, the specific role of IL-33/ST2 axis that might predominate in tumor remains to be further studied. Ongoing phase II clinical studies (NCT03267316 and NCT05116891) on IL-1RAcP antibody CAN04, which showed the potency of selective cytokine signaling inhibition of IL-1 family cytokines in NSCLC (62), may hopefully find a new method to treat lung cancer.

## The Role and Mechanism of IL-33 in Lung Cancer

### The Promoting Effect of IL-33 on Pulmonary Fibrosis and Lung Cancer

Lung cancer is a common intrapulmonary complication found in patients with pulmonary fibrosis, so the mechanisms that can promote pulmonary fibrosis are likely to simultaneously contribute to the development of lung cancer. For instance, it is reported that the interaction between IL-33 and ST2 on airway epithelial cells stimulated by house dust mite (HDM) under asthma conditions can promote the expression of cluster of differentiation 146 (CD146) and further amplify the inflammatory response, epithelial-mesenchymal transition (EMT) and airway remodeling (63). In pulmonary fibrosis, IL-33 was able to polarize M2 macrophages to produce IL-13 and TGF-β_1_, and could induce ILC2 to produce IL-13 (64). TGF-β and IL-13 can induce myofibroblast differentiation and stimulate the production of extracellular matrix components, which contribute to pulmonary fibrosis (65, 66). Moreover, it is found that Akt1 (Akt is also known as protein kinase B) as well as Akt2 are involved in the IL-33-induced secretion of IL-13 by macrophage, while Akt2 also took part in IL-33-induced secretion of TGF-β (67, 68). Yi, X., et al. (69) reported that ubiquitin-specific protease 38 (USP 38) was able to cause the autophagic degradation of ST2, thereby inhibiting IL-33 signaling and attenuating bleomycin-induced pulmonary fibrosis. In summary, the current studies have established that IL-33 can play a pivotal role in promoting the process of pulmonary fibrosis. Thus, the application of IL-33 inhibitors or enhancement of mast cells and fibroblasts to produce the decoy receptor sST2 might significantly slow the progression of pulmonary fibrosis development, and inhibiting this process can help to reduce the possibility of pulmonary fibrosis patients developing lung cancer.

**Figure 1:**
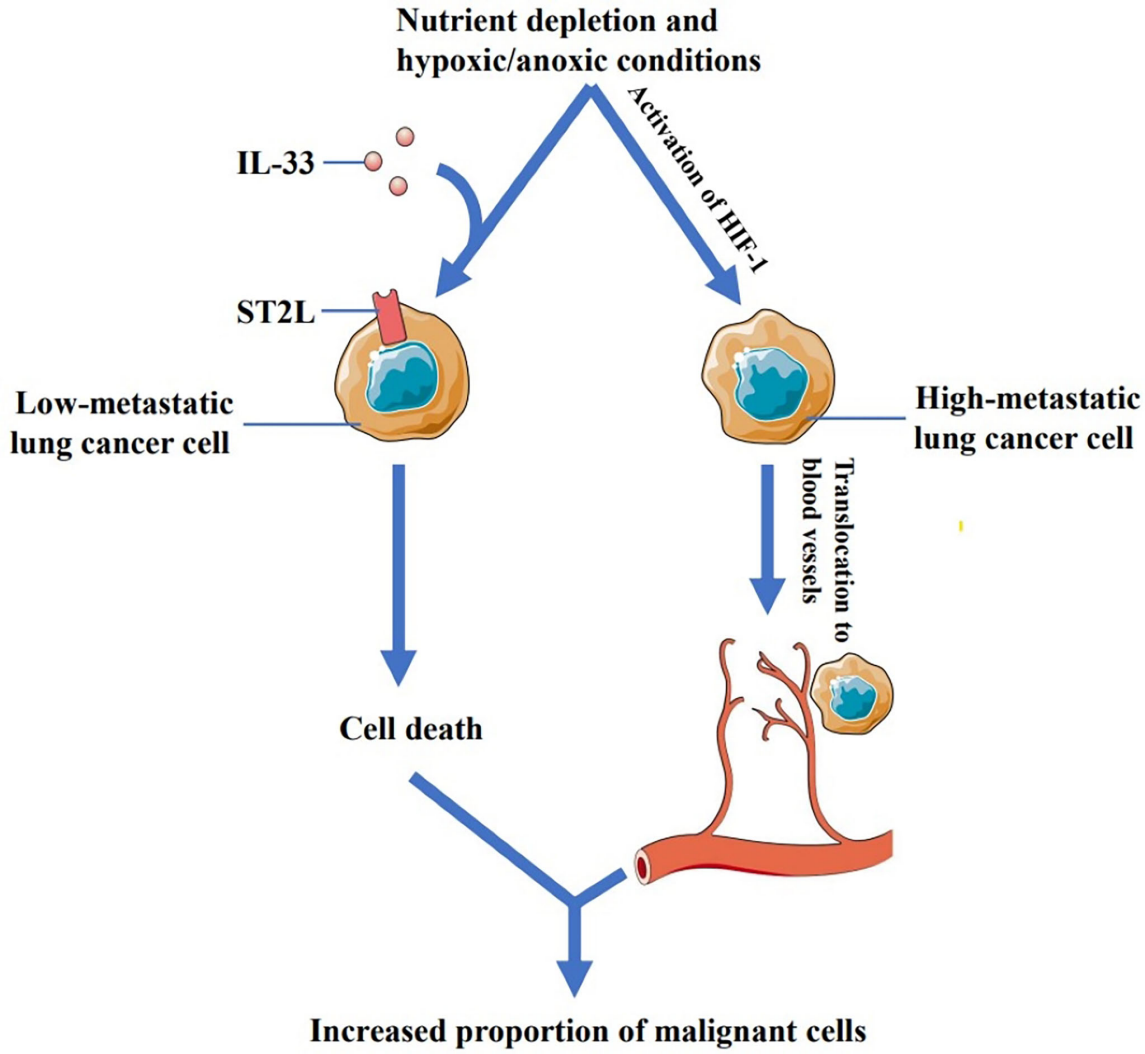
Low-metastatic cells derived from Lewis lung carcinoma express ST2L while high-metastatic cells do not. IL-33 can increase the cell death in low metastatic Lewis lung cancer cells under nutrient depletion and hypoxic/anoxic conditions, while ST2L-negative high-metastatic lung cancer cells are not affected by IL-33. IL-33 thus provides a selective pressure for ST2L-negative cells that might expand into regions adjacent to the tumor blood vessels following hypoxia-inducible factor 1 (HIF-1)-dependent translocation, thereby increasing the proportion of the malignant cells.

Akimoto, M., et al. (70) found that low-metastatic cells derived from Lewis lung carcinoma expressed functional ST2L while high-metastatic cells did not. Their study also found that recombinant IL-33 (rIL-33) increased the cell death in low-metastatic Lewis lung cancer cells under nutrient depletion and hypoxic/anoxic conditions. Interestingly, IL-1β-induced IL-33 was also involved in promoting the death of low-metastatic cells. ST2L-negative highly metastatic lung cancer cells were found to be insensitive to IL-33. IL-33 thus provides a selective pressure for ST2L-negative cells that might expand into regions adjacent to the tumor blood vessels following hypoxia-inducible factor 1 (HIF-1)-dependent translocation (71), thereby increasing the proportion of the malignant cells (**Figure 1**). Moreover, another study by Babadi AS et al. (72) showed that IL-33-ST2 signaling resulted in programmed death of low-metastatic Lewis lung cancer cells, whereas high-metastatic cells were not substantially affected, which eventually facilitated tumor growth. Therefore, Akimot et al. concluded that anti-IL-33 antibody or sST2 may be useful for inhibiting malignant progression and alleviating lung cancer.

It is reported that up-regulating the expression level of IL-33 increased the malignant growth of lung cancer tumor cells *in vitro*. They also found that the tumor volume of lung cancer tumor cells transfected with IL-33 expression vector increased significantly in immunodeficient mice (73). Their research findings further indicated that the interaction between IL-33 and ST2 could up-regulate the level of glucose transporter type 1 (GLUT1) in the cell membrane, thereby enhancing the glucose uptake and glycolysis of lung cancer cells, and providing optimal energy support for the malignant growth of lung cancer cells. They concluded that enhancing IL-33/ST2 signaling was able to markedly promote the malignant growth of lung cancer cells, while down-regulating or blocking IL-33/ST2 signaling could inhibit this process.

**Figure 2:**
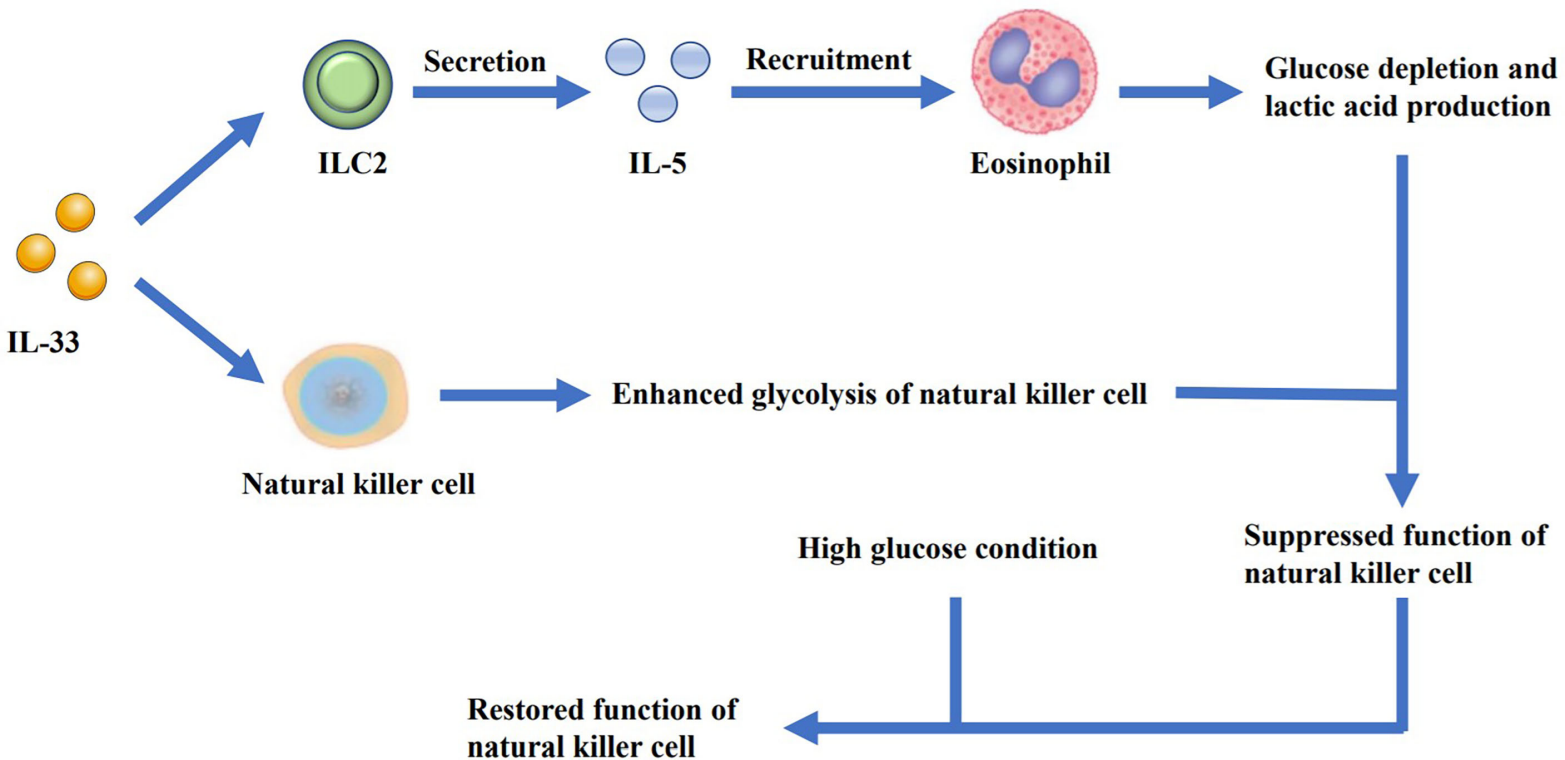
IL-33 promotes the secretion of IL-5 by ILC2s and leads to the recruitment of eosinophils, resulting in glucose depletion and lactic acid production. At the same time, IL-33 can enhance the glycolysis of NK cells. As the glucose is depleted and the lactic acid is accumulated, the function of NK cells is suppressed. However, when additional glucose is added, the function of the cells can be restored.

Jiang, Y., et al. (74) found that IL-33 increased the secretion of the antimicrobial peptide LL-37 in macrophages. LL-37 cooperated with IL-33 to increase the phosphorylation of p38 MAPK as well as NF-κB p65 pathways, and augmented IL-6 and IL-1β secretion, thus resulting in the proliferation of lung cancer cells *in vitro*. Zhou, X. et al. (75) reported that IL-33 inhibited the expression of cell death-inducing p53 target protein 1 (CDIP1) by regulating the miR-128-3p/CDIP1 signaling pathway, thereby effectively reducing the apoptosis of NSCLC cells. Yang, Z. et al. (57) found that IL-33 treatment promoted the lung cancer cell migration as well as invasion and increased the expression of matrix metalloproteinase-2 (MMP2) and matrix metalloproteinase-9 (MMP9) in a ST2-dependent manner. The AKT signaling pathway was also found to be involved in this process. A study by Wang, K. et al. (76) further demonstrated that NSCLC-derived IL-33, which caused immune escape of the tumor cells, supported tumor growth. IL-33 exerted a positive effect on FoxP3 induction, thus increasing the frequency of Tregs in the tumor-infiltrating lymphocytes, which was partially dependent on M2 TAMs (57). Another study on Kras-mutant mice showed that treatment with anti-ST2 antibody could reduce Tregs in tumor, which led to restoration of NK cell activity and enhanced Th1 activity, with increased CD8^+^ T cell response (77). It is also reported that relying on IL-5-induced lung eosinophilia, IL-33-dependent ILC2 activation could suppress the metabolic fitness of NK cells (**Figure 2**), which could be reversed by high glucose condition (78). In conclusion, IL-33 can potentially enhance the malignant growth ability of lung cancer tumor cells under specific conditions.

### The Inhibitory Effect of IL-33 on Lung Cancer Progression and Metastasis

Li, (79) used human peripheral blood to confirm that IL-33 can promote the secretion of IFN-γ and GzmB in PBMCs *in vitro*, and further confirmed that IL-33 can enhance the cytotoxic effect of PBMCs on tumor cells and increase the activity of PMBCs to eliminate the lung cancer cells. They also found that IL-33 was able to downregulate PD-1 expression on CD3-positive and CD8-positive cell subsets in PMBCs, thereby improving the function of CD8^+^ T cells in immune responses and reducing T cell exhaustion, which was consistent with the findings of Liang, Y. et al. (80).

It is reported that IL-33 promoted the proliferation and activation of CD8^+^ T cells and NK cells through modulating the NF-κB signaling pathway, and promoted their infiltration in the tumor lung metastasis, thereby producing a significant effect of inhibiting tumor growth and lung metastasis (81). Qi, L. et al. (82) found that IL-33 was able to activate and recruit NK cells in a TNF-α-dependent manner to inhibit the development of lung metastases, which was consistent with the findings of previous studies. It is found that IL-33 induced cylindromatosis (CYLD)-activated DCs, thus inducing T cell proliferation and IL-17 secretion in lung adenocarcinoma and thereby enhanced antitumor effects (83). In addition, it is reported that IL-33 could promote the differentiation and maturation of DCs, which augmented the antitumor effect of CD8^+^ T cells and NK cells and inhibited the proliferation of lung cancer cells through affecting MyD88 pathway (84).

Moreover, mechanistic studies by Liang, Y. et al. (80) indicated that IL-33 treatment increased the expression of GLUT1 and increased the transcription of several glycolytic enzymes in CD8^+^ T cells. Their findings also suggested that IL-33 might promote CD8 effector T cell responses by activating mTORC1 and driving aerobic glycolysis. This mechanism might effectively help to inhibit both the tumor growth and metastasis.

In conclusion, IL-33 can inhibit lung tumor metastasis and tumor cell proliferation by increasing the proliferation and activation of immune cells and promoting their infiltration into various tissues.

### The Relationship Between the Level of IL-33 and Progression of Lung Cancer

Akimoto et al. (70) found that the expression level of IL-33 in the tumor tissue of lung cancer patients gradually decreased with an increase of the clinical grade of the cancer. It is reported that the expression of IL-33 in the tumor tissue samples of NSCLC patients significantly decreased compared with that in adjacent tissue (85). A study selected 125 NSCLC patients and found that patients with relatively higher expression of IL-33 in the paracancerous tissue were more likely to develop the poorly differentiated lung cancer and lymph node metastasis (86). Hu, L. et al. (87) analyzed 262 NSCLC patients, and found that the serum IL-33 level of the patients could be significantly higher than that of the healthy control. They also concluded that baseline serum IL-33 was an independent prognostic factor in NSCLC patients. However,Yang, M. et al. (88) found in a study of surgically resected lung cancer tissue and adjacent normal tissue specimens from 127 NSCLC patients that relatively higher IL-33 expression in the tumor was associated with longer survival in patients with NSCLC adenocarcinoma but not in squamous cell carcinoma. They also observed that in adenocarcinoma and squamous cell carcinoma, the expression level of IL-33 in tumor tissues was significantly lower as compared with its adjacent normal tissue, which was consistent with the findings of Feng, Y. et al. In conclusion, the concentration of IL-33 in the tumor was reported to be lower than that in the paracancerous tissue, and the high level of serum IL-33 in NSCLC patients was associated with tumor malignancy, but the high level of IL-33 in lung adenocarcinoma tumor tissue could predict longer survival time.

In a recent study, which used mouse model of squamous cell carcinoma, Taniguchi, S. et al. (89) found IL-33-TGF-β niche signaling loops between tumor-initiating cells (TICs) and FcϵRIα^+^ macrophages, thus indicating that IL-33 can aid to create a microenvironment that can promote the tumor growth, and whether this mechanism exists in lung cancer remains to be further investigated. In summary, if the IL-33-TGF-β niche signaling loop is also present in lung cancer, it will be necessary to investigate that why IL-33 levels were relatively lower in lung cancer tumor tissues. In addition, whether a specific mechanism allows the tumor cells to proliferate to a certain level before it stops releasing pro-proliferative substances by itself, thereby reducing the death of tumor cells caused by insufficient nutrient supply, and finally showing the phenomenon of stable growth of tumors in advanced lung cancer needs to be further analyzed.

## The Relationship Between Anti-Lung Cancer Drugs and IL-33

### EGFR Inhibitor Gefitinib and IL-33

Both EGFR and HER2 have been reported to be involved in autonomous cell proliferation and clonal expansion, accelerated intraepithelial proliferation, growth factor (GF)-induced basement membrane rupture as well as invasive growth, apoptosis evasion, and vascular growth in tumor progression (90). EGFR is frequently mutated and overexpressed in various cancer cells, especially non-small cell lung cancer. Mutated EGF tyrosine kinase receptor is constitutively expressed, thereby resulting in uncontrolled cell growth and proliferation. It has been established that by inhibiting this growth factor receptor, gefitinib can block the intracellular Ras signaling cascade, thereby suppressing the growth of the malignant cells (91). The U.S. FDA has already approved gefitinib for the treatment of NSCLC (3).

The effects of EGFR inhibition include impaired growth and migration of keratinocytes, as well as inflammatory chemokine expression by these cells (92). These effects lead to inflammatory cell recruitment and subsequent skin damage (93). A number of studies have also shown that transgenic mouse models overexpressing IL-31 develop severe pruritus with alopecia and skin lesions such as hyperkeratosis, acanthosis, inflammatory cell infiltration, as well as mastocytosis (94). The receptors for IL-31, IL-31RA, and the receptor for IL-33, ST2, are commonly expressed on dermal fibroblasts, and it is speculated that IL-31 and IL-33 can synergistically stimulate basophils that interact with fibroblasts to release atopic dermatitis (AD)-related chemokines (95). Combining the above research results, Sebastiano Gangemi et al. (96) based on the study of patients treated with gefitinib, proposed that EGFR-TK inhibitors can cause extensive keratinocyte damage, thereby resulting in the release of IL-33, which in turn can interact with the receptors on mast cells, thus stimulating the secretion of several cytokines, including IL-31, that can cause skin manifestations. We assume that downregulating the expression of IL-33 may alleviate the substantial skin damage caused by gefitinib treatment.

### Topo II Inhibitor Doxorubicin and IL-33

There are two distinct mechanisms of action of doxorubicin in cancer cells: DNA intercalation and destruction of topoisomerase-II-mediated DNA repair and generation of free radicals and their damage to cell membranes, DNA and proteins (97). Doxorubicin can be oxidized to semiquinone, an unstable metabolite, which in turn is converted to doxorubicin during the release of reactive oxygen species (ROS). ROS can cause extensive lipid peroxidation and membrane damage, DNA damage, oxidative stress, and trigger apoptotic pathways of cell death (98). It has been approved by the U.S. FDA for the treatment of both NSCLC and SCLC (3, 99).

Deng, Y. et al. (100) reported that ILC2 can expand and produce IL-4 immediately before activation of macrophages, dendritic cells and IL-4^+^ T after doxorubicin-induced myocardial necrosis. They speculated that in response to myocardial injury, cardiac ILC2s act as first-line responders and produce IL-4 to promote the responses during inflammation and cardiac tissue repair. While IL-33 can promote the production of IL-4 by Th2 cells (42), they proposed that IL-33-triggered ILC2 responses have the potential to alleviate cardiac injury from doxorubicin.

Among the kinases activated by ROS, ERK, p38 and JNK have been shown to play key roles in the process of oxidative apoptosis (101, 102). Yao, Y. et al. (103) found that doxorubicin could increase the phosphorylation of ERK, p38 and JNK, while IL-33 inhibited JNK activation. Moreover, other studies have also found that co-treatment with IL-33 did not affect ERK phosphorylation, but reduced the angiotensin II-induced p38 and JNK phosphorylation (104). In addition, targeted inhibition of JNK can attenuate doxorubicin-induced cardiomyocyte injury (105). Taken together, they proposed that IL-33 can protect the cardiomyocytes from doxorubicin-induced cardiomyocyte apoptosis by inhibiting the ASK1/JNK signaling pathway. Sabapathy, V. et al. (106) reported in a model of doxorubicin-induced nephrotoxic kidney injury that IL-233 (a molecule with the activity of IL-2 and IL-33) can potentiate Treg and ILC2 to inhibit renal injury while promoting repair. In conclusion, IL-33 combined with doxorubicin might reduce the cardiac damage and kidney injury observed during the lung cancer treatment.

## Conclusion

**Figure 3:**
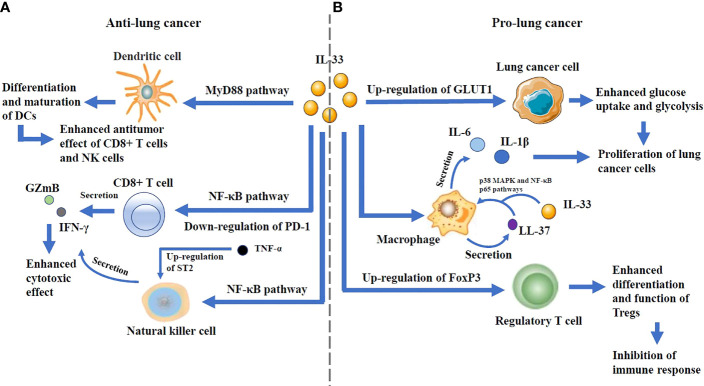
**(A)** The anti-lung cancer effects: IL-33 can promote the differentiation and maturation of DCs through MyD88 pathway, thus increasing the antitumor effect of CD8+ T cells and NK cells. It is also able to enhance the secretion of GZmB and IFN-γ by CD8+ T cells and NK cells through NF-κB pathway. Notably, IL-33 downregulates PD-1 expression in CD8+ T cells, and its interaction with TNF-α, which up-regulates ST2 expression in NK cells, enhances antitumor effects. **(B)** The pro-lung cancer effects: By up-regulating the expression of GLUT1 on lung cancer cells, IL-33 can enhance their glucose uptake and glycolysis, which lead to the proliferation of lung cancer cells. IL-33 increases the secretion of the antimicrobial peptide LL-37 in macrophages. LL-37 cooperates with IL-33 to increase the phosphorylation of p38 MAPK as well as NF-kB p65 pathways, and augment IL-6 and IL-1b secretion, thus resulting in the proliferation of lung cancer cells. IL-33 can increase the frequency of Tregs in tumor-infiltrating lymphocytes and enhance the differentiation and function of Tregs by up-regulating the expression of FoxP3, which can lead to immune escape.

IL-33 can play a dual role and has been reported to promote lung cancer in some studies and suppress lung cancer in others (**Figure 3**).

The possible mechanisms of pro-lung cancer effects could be explained by the findings that IL-33 can promote EMT by increasing the expression of CD146 in airway epithelial cells, induce ILC2 to produce IL-13, and polarize M2 macrophages to generate IL-13 and TGF-β (64), these factors can promote pulmonary fibrosis, which might induce lung cancer. rIL-33 increases the death of low-metastatic Lewis lung cancer cells under nutrient depletion and hypoxic/anoxic conditions, while the surviving highly metastatic cells might expand to the area near the tumor blood vessels after HIF-1-dependent translocation and promote the development of lung cancer. It has been reported that in Lewis lung cancer, IL-33 can increase the secretion of the antimicrobial peptide LL-37 from macrophages, resulting in excessive inflammation that can facilitate the tumor growth. NSCLC cells secrete IL-33 to increase the frequency of Tregs in tumor-infiltrating lymphocytes, which can lead to immune escape. By activating ILC2s, which leads to the recruitment of eosinophils to lung, IL-33 can suppress the function of NK cells. IL-33 secreted by cancer-associated fibroblasts is able to recruit inflammatory cells into the metastatic microenvironment, which facilitates the lung metastasis of breast cancer (107). IL-33 treatment is also reported to induce a population of circulating inflammatory ILC2s and inhibit type 1 immunity against multiple myeloma (108).

On the contrary, to exert its potential anti-lung cancer mechanism IL-33 can promote the secretion of IFN-γ and GZmB in PBMCs, thereby enhancing the cytotoxic effect of PBMCs on lung cancer cells, and downregulate PD-1 expression on CD3 positive and CD8 positive cell subsets in PMBCs, thereby improving the function of CD8^+^ T cells in immune responses. In lung adenocarcinoma model, IL-33 can activate NK in a TNF-α-dependent manner through activating the NF-κB signaling pathway, and promoting the proliferation and activation of CD8^+^ T cells, consequently inhibiting tumor metastasis. IL-33 may stimulate CD8^+^ T cell mediated responses by activating mTORC1 and promoting aerobic glycolysis. IL-33 can induce CYLD-activated DCs in mouse lung adenocarcinomas, thereby inducing the proliferation of T cells and the secretion of IL-17 to eradicate the tumors. It has also been reported that in melanoma and colon cancer models IL-33 can effectively promote the function of immune cells to inhibit cancer cells from metastasizing (109, 110), which suggests that the antitumor effect of IL-33 may also be predominant in lung cancer. While a study reported that IL-33-induced activation of ILC2s may lead to the reduced function of lung NK cells, others found that ILC2s could interact with DCs and T cells to augment the antitumor function in a variety of cancers including lung cancer (111, 112).

Many clinical studies show that the high expression of IL-33 in the paracancerous tissues of NSCLC patients and its high level in the serum indicate higher tumor malignancy and poor prognosis. However, the high level of IL-33 in the tumor tissue of lung adenocarcinoma can lead to relatively longer survival period, thus indicating that IL-33 may play different roles in different types of lung cancer tissue. In addition, studies have also shown that IL-33 level in tumor tissues of patients with NSCLC is significantly lower than that in adjacent tissues. Is the higher level of serum IL-33 in patients with more aggressive lung cancer a vicious circle in which the surrounding tissues are stimulated to secrete IL-33 to promote the tumor growth, or is it a protection against a large number of tumor cells and their rapid proliferation that exerts a substantial anti-tumor effect. The mechanism still remains to be studied.

According to the existing results, we assume that the level of Tregs in the tumor tissue is an important determinant of IL-33 function in lung cancer. The less Tregs in TME, the stronger the antitumor effect of IL-33. With the formation of tumor-promoting microenvironment and the activation of inhibitory immune cells, the antitumor effect of IL-33 is attenuated or even reversed. Blocking the IL-33/ST2 signaling can serve as a potential lung cancer therapeutic mechanism that can effectively inhibit the proliferation and migration of lung cancer cells.

## Author Contributions

KY designed and drafted the manuscript; CT and CZ edited the manuscript; MX designed and edited the manuscript. All authors contributed to the article and approved the submitted version.

## Funding

This work was supported by the National Natural Science Foundation of China (No. 81872000 and No. 81670521).

## Conflict of Interest

The authors declare that the research was conducted in the absence of any commercial or financial relationships that could be construed as a potential conflict of interest.

## Publisher’s Note

All claims expressed in this article are solely those of the authors and do not necessarily represent those of their affiliated organizations, or those of the publisher, the editors and the reviewers. Any product that may be evaluated in this article, or claim that may be made by its manufacturer, is not guaranteed or endorsed by the publisher.

## References

[1] Cayrol C. IL-33, an Alarmin of the IL-1 Family Involved in Allergic and Non Allergic Inflammation: Focus on the Mechanisms of Regulation of Its Activity. Cells (2021) 11(1):107. doi: 10.3390/cells11010107 35011670 PMC8750818

[2] Siegel RL Miller KD Fuchs HE Jemal A. Cancer Statistic. CA Cancer J Clin (2021) 71:7–33. doi: 10.3322/caac.21654 33433946

[3] Board P. D. Q. A. T. E. Non-Small Cell Lung Cancer Treatment (PDQ®): Patient Version. In: PDQ Cancer Information Summaries. Bethesda (MD: National Cancer Institute (US (2021).

[4] Ferrantini M Capone I Belardelli F. Interferon-Alpha and Cancer: Mechanisms of Action and New Perspectives of Clinical Use. Biochimie (2007) 89:884–93. doi: 10.1016/j.biochi.2007.04.006 17532550

[5] Lu J Kang J Zhang C Zhang X. The Role of IL-33/ST2L Signals in the Immune Cells. Immunol Lett (2015) 164:11–7. doi: 10.1016/j.imlet.2015.01.008 25662624

[6] Little CD Nau MM Carney DN Gazdar AF Minna JD. Amplification and Expression of the C-Myc Oncogene in Human Lung Cancer Cell Lines. Nature (1983) 306:194–6. doi: 10.1038/306194a0 6646201

[7] Nau MM Brooks BJ Battey J Sausville E Gazdar AF Kirsch IR. L-Myc, a New Myc-Related Gene Amplified and Expressed in Human Small Cell Lung Cancer. Nature (1985) 318:69–73. doi: 10.1038/318069a0 2997622

[8] Nau MM Carney DN Battey J Johnson B Little C Gazdar A. Amplification, Expression and Rearrangement of C-Myc and N-Myc Oncogenes in Human Lung Cancer. Curr Top Microbiol Immunol (1984) 113:172–7. doi: 10.1007/978-3-642-69860-6_29 6090062

[9] Sher T Dy GK Adjei AA. Small Cell Lung Cancer. Mayo Clin Proc (2008) 83:355–67. doi: 10.4065/83.3.355 18316005

[10] Rudin CM Brambilla E Faivre-Finn C Sage J. Small-Cell Lung Cancer. Nat Rev Dis Primers (2021) 7:3. doi: 10.1038/s41572-020-00235-0 33446664 PMC8177722

[11] Wistuba Ii Gazdar AF Minna JD. Molecular Genetics of Small Cell Lung Carcinoma. Semin Oncol (2001) 28:3–13. doi: 10.1016/S0093-7754(01)90072-7 11479891

[12] Osmani L Askin F Gabrielson E Li QK. Current WHO Guidelines and the Critical Role of Immunohistochemical Markers in the Subclassification of non-Small Cell Lung Carcinoma (NSCLC): Moving From Targeted Therapy to Immunotherapy. Semin Cancer Biol (2018) 52:103–9. doi: 10.1016/j.semcancer.2017.11.019 PMC5970946 29183778

[13] Khullar OV Liu Y Gillespie T Higgins KA Ramalingam S Lipscomb J. Survival After Sublobar Resection Versus Lobectomy for Clinical Stage IA Lung Cancer: An Analysis From the National Cancer Data Base. J Thorac Oncol (2015) 10:1625–33. doi: 10.1097/JTO.0000000000000664 PMC5798611 26352534

[14] Billmeier SE Ayanian JZ Zaslavsky AM Nerenz DR Jaklitsch MT Rogers SO. Predictors and Outcomes of Limited Resection for Early-Stage non-Small Cell Lung Cancer. J Natl Cancer Inst. (2011) 103:1621–9. doi: 10.1093/jnci/djr387 PMC3206042 21960708

[15] Kang J Zhang C Zhong WZ. Neoadjuvant Immunotherapy for non-Small Cell Lung Cancer: State of the Art. Cancer Commun (Lond) (2021) 41:287–302. doi: 10.1002/cac2.12153 33689225 PMC8045926

[16] Duma N Santana-Davila R Molina JR. Non-Small Cell Lung Cancer: Epidemiology, Screening, Diagnosis, and Treatment. Mayo Clin Proc (2019) 94:1623–40. doi: 10.1016/j.mayocp.2019.01.013 31378236

[17] Evan GI Vousden KH. Proliferation, Cell Cycle and Apoptosis in Cancer. Nature (2001) 411:342–8. doi: 10.1038/35077213 11357141

[18] Byers LA Rudin CM. Small Cell Lung Cancer: Where do We Go From Here? Cancer (2015) 121:664–72. doi: 10.1002/cncr.29098 PMC5497465 25336398

[19] Wang Y Zou S Zhao Z Liu P Ke C Xu S. New Insights Into Small-Cell Lung Cancer Development and Therapy. Cell Biol Int (2020) 44:1564–76. doi: 10.1002/cbin.11359 PMC7496722 32281704

[20] Altan M Chiang AC. Management of Small Cell Lung Cancer: Progress and Updates. Cancer J (2015) 21:425–33. doi: 10.1097/PPO.0000000000000148 26389768

[21] Aupérin A Arriagada R Pignon JP Le Péchoux C Gregor A Stephens RJ. Prophylactic Cranial Irradiation for Patients With Small-Cell Lung Cancer in Complete Remission. Prophylactic Cranial Irradiation Overview Collaborative Group. N Engl J Med (1999) 341:476–84. doi: 10.1056/NEJM199908123410703 10441603

[22] Slotman B Faivre-Finn C Kramer G Rankin E Snee M Hatton M. Prophylactic Cranial Irradiation in Extensive Small-Cell Lung Cancer. N Engl J Med (2007) 357:664–72. doi: 10.1056/NEJMoa071780 17699816

[23] Takahashi T Yamanaka T Seto T Harada H Nokihara H Saka H. Prophylactic Cranial Irradiation Versus Observation in Patients With Extensive-Disease Small-Cell Lung Cancer: A Multicentre, Randomised, Open-Label, Phase 3 Trial. Lancet Oncol (2017) 18:663–71. doi: 10.1016/S1470-2045(17)30230-9 28343976

[24] Palma DA Nguyen TK Louie AV Malthaner R Fortin D Rodrigues GB. Measuring the Integration of Stereotactic Ablative Radiotherapy Plus Surgery for Early-Stage Non-Small Cell Lung Cancer: A Phase 2 Clinical Trial. JAMA Oncol (2019) 5:681–8. doi: 10.1001/jamaoncol.2018.6993 PMC6512269 30789648

[25] Lagerwaard FJ Haasbeek CJ Smit EF Slotman BJ Senan S. Outcomes of Risk-Adapted Fractionated Stereotactic Radiotherapy for Stage I non-Small-Cell Lung Cancer. Int J Radiat Oncol Biol Phys (2008) 70:685–92. doi: 10.1016/j.ijrobp.2007.10.053 18164849

[26] Satouchi M Nosaki K Takahashi T Nakagawa K Aoe K Kurata T. First-Line Pembrolizumab vs Chemotherapy in Metastatic non-Small-Cell Lung Cancer: KEYNOTE-024 Japan Subset. Cancer Sci (2021) 112:5000–10. doi: 10.1111/cas.15144 PMC8645705 34543477

[27] Sequist LV Yang JC Yamamoto N O'byrne K Hirsh V Mok T. Phase III Study of Afatinib or Cisplatin Plus Pemetrexed in Patients With Metastatic Lung Adenocarcinoma With EGFR Mutations. J Clin Oncol (2013) 31:3327–34. doi: 10.1200/JCO.2012.44.2806 23816960

[28] Lee CK Wu YL Ding PN Lord SJ Inoue A Zhou C. Impact of Specific Epidermal Growth Factor Receptor (EGFR) Mutations and Clinical Characteristics on Outcomes After Treatment With EGFR Tyrosine Kinase Inhibitors Versus Chemotherapy in EGFR-Mutant Lung Cancer: A Meta-Analysis. J Clin Oncol (2015) 33:1958–65. doi: 10.1200/JCO.2014.58.1736 25897154

[29] Zhang Y Zhou H Zhang L. Which is the Optimal Immunotherapy for Advanced Squamous non-Small-Cell Lung Cancer in Combination With Chemotherapy: Anti-PD-1 or Anti-PD-L1? J Immunother Cancer (2018) 6:135. doi: 10.1186/s40425-018-0427-6 30509312 PMC6276157

[30] Cayrol C Girard JP. IL-33: An Alarmin Cytokine With Crucial Roles in Innate Immunity, Inflammation and Allergy. Curr Opin Immunol (2014) 31:31–7. doi: 10.1016/j.coi.2014.09.004 25278425

[31] Roussel L Erard M Cayrol C Girard JP. Molecular Mimicry Between IL-33 and KSHV for Attachment to Chromatin Through the H2A-H2B Acidic Pocket. EMBO Rep (2008) 9:1006–12. doi: 10.1038/embor.2008.145 PMC2572127 18688256

[32] Lefrançais E Roga S Gautier V Gonzalez-De-Peredo A Monsarrat B Girard JP. IL-33 is Processed Into Mature Bioactive Forms by Neutrophil Elastase and Cathepsin G. Proc Natl Acad Sci USA (2012) 109(5):1673–8. doi: 10.1073/pnas.1115884109 PMC3277172 22307629

[33] Lefrançais E Duval A Mirey E Roga S Espinosa E Cayrol C. Central Domain of IL-33 is Cleaved by Mast Cell Proteases for Potent Activation of Group-2 Innate Lymphoid Cells. Proc Natl Acad Sci USA (2014) 111(43):15502–7. doi: 10.1073/pnas.1410700111 PMC4217470 25313073

[34] Schmitz J Owyang A Oldham E Song Y Murphy E Mcclanahan TK. IL-33, an Interleukin-1-Like Cytokine That Signals *via* the IL-1 Receptor-Related Protein ST2 and Induces T Helper Type 2-Associated Cytokines. Immunity (2005) 23:479–90. doi: 10.1016/j.immuni.2005.09.015 16286016

[35] Lingel A Weiss TM Niebuhr M Pan B Appleton BA Wiesmann C. Structure of IL-33 and its Interaction With the ST2 and IL-1racp Receptors–Insight Into Heterotrimeric IL-1 Signaling Complexes. Structure (2009) 17:1398–410. doi: 10.1016/j.str.2009.08.009 PMC2766095 19836339

[36] Byers DE Alexander-Brett J Patel AC Agapov E Dang-Vu G Jin X. Long-Term IL-33-Producing Epithelial Progenitor Cells in Chronic Obstructive Lung Disease. J Clin Invest (2013) 123:3967–82. doi: 10.1172/JCI65570 PMC3754239 23945235

[37] Préfontaine D Nadigel J Chouiali F Audusseau S Semlali A Chakir J. Increased IL-33 Expression by Epithelial Cells in Bronchial Asthma. J Allergy Clin Immunol (2010) 125:752–4. doi: 10.1016/j.jaci.2009.12.935 20153038

[38] Préfontaine D Lajoie-Kadoch S Foley S Audusseau S Olivenstein R Halayko AJ. Increased Expression of IL-33 in Severe Asthma: Evidence of Expression by Airway Smooth Muscle Cells. J Immunol (2009) 183:5094–103. doi: 10.4049/jimmunol.0802387 19801525

[39] Naumnik W Naumnik B Niewiarowska K Ossolinska M Chyczewska E. Novel Cytokines: IL-27, IL-29, IL-31 and IL-33. Can They be Useful in Clinical Practice at the Time Diagnosis of Lung Cancer? Exp Oncol (2012) 34:348–53. https://exp-oncology.com.ua/article/4001 23302994

[40] Hu LA Fu Y Zhang DN Zhang J. Serum IL-33 as a Diagnostic and Prognostic Marker in non- Small Cell Lung Cancer. Asian Pac. J Cancer Prev (2013) 14(4):2563–6. doi: 10.7314/apjcp.2013 23725175

[41] Kim MS Kim E Heo JS Bae DJ Lee JU Lee TH. Circulating IL-33 Level is Associated With the Progression of Lung Cancer. Lung Cancer (2015) 90:346–51. doi: 10.1016/j.lungcan.2015.08.011 26342550

[42] Millar NL O'donnell C Mcinnes IB Brint E. Wounds That Heal and Wounds That Don't - The Role of the IL-33/ST2 Pathway in Tissue Repair and Tumorigenesis. Semin Cell Dev Biol (2017) 61:41–50. doi: 10.1016/j.semcdb.2016.08.007 27521518

[43] Kakkar R Hei H Dobner S Lee RT. Interleukin 33 as a Mechanically Responsive Cytokine Secreted by Living Cells. J Biol Chem (2012) 287:6941–8. doi: 10.1074/jbc.M111.298703 PMC3307313 22215666

[44] Kouzaki H Iijima K Kobayashi T O'grady SM Kita H. The Danger Signal, Extracellular ATP, is a Sensor for an Airborne Allergen and Triggers IL-33 Release and Innate Th2-Type Responses. J Immunol (2011) 186:4375–87. doi: 10.4049/jimmunol.1003020 PMC3062674 21357533

[45] Uchida M Anderson EL Squillace DL Patil N Maniak PJ Iijima K. Oxidative Stress Serves as a Key Checkpoint for IL-33 Release by Airway Epithelium. Allergy (2017) 72:1521–31. doi: 10.1111/all.13158 PMC5591045 28273344

[46] Zhou W Zhang J Toki S Goleniewska K Norlander AE Newcomb DC. COX Inhibition Increases Alternaria-Induced Pulmonary Group 2 Innate Lymphoid Cell Responses and IL-33 Release in Mice. J Immunol (2020) 205:1157–66. doi: 10.4049/jimmunol.1901544 PMC7644167 32690653

[47] Drake LY Kita H. IL-33: Biological Properties, Functions, and Roles in Airway Disease. Immunol Rev (2017) 278:173–84. doi: 10.1111/imr.12552 PMC5492954 28658560

[48] Schiering C Krausgruber T Chomka A Fröhlich A Adelmann K Wohlfert EA. The Alarmin IL-33 Promotes Regulatory T-Cell Function in the Intestine. Nature (2014) 513:564–8. doi: 10.1038/nature13577 PMC4339042 25043027

[49] Ercolano G Gomez-Cadena A Dumauthioz N Vanoni G Kreutzfeldt M Wyss T. PPARɣ; Drives IL-33-Dependent ILC2 Pro-Tumoral Functions. Nat Commun (2021) 12:2538. doi: 10.1038/s41467-021-22764-2 33953160 PMC8100153

[50] Mai S Liu L Jiang J Ren P Diao D Wang H. Oesophageal Squamous Cell Carcinoma-Associated IL-33 Rewires Macrophage Polarization Towards M2 *via* Activating Ornithine Decarboxylase. Cell Prolif. (2021) 54:e12960. doi: 10.1111/cpr.12960 33305406 PMC7848962

[51] Wang J Zhong M Liu J. Research Progress on the Biological Function of IL-33 and its Effect on Tumors. J Biol (2018) 35:76–9. doi: 10.3969/j.issn.2095-1736.2018.03.076

[52] Tominaga S Kuroiwa K Tago K Iwahana H Yanagisawa K Komatsu N. Presence and Expression of a Novel Variant Form of ST2 Gene Product in Human Leukemic Cell Line UT-7/GM. Biochem Biophys Res Commun (1999) 264:14–8. doi: 10.1006/bbrc.1999.1469 10527832

[53] Iwahana H Hayakawa M Kuroiwa K Tago K Yanagisawa K Noji S. Molecular Cloning of the Chicken ST2 Gene and a Novel Variant Form of the ST2 Gene Product, ST2LV. Biochim Biophys Acta (2004) 1681:1–14. doi: 10.1016/j.bbaexp.2004.08.013 15566939

[54] Griesenauer B Paczesny S. The ST2/IL-33 Axis in Immune Cells During Inflammatory Diseases. Front Immunol (2017) 8:475. doi: 10.3389/fimmu.2017.00475 28484466 PMC5402045

[55] Larsen KM Minaya MK Vaish V Peña MMO. The Role of IL-33/ST2 Pathway in Tumorigenesis. Int J Mol Sci (2018) 19(9):2676. doi: 10.3390/ijms19092676 PMC6164146 30205617

[56] Zarezadeh Mehrabadi A Aghamohamadi N Khoshmirsafa M Aghamajidi A Pilehforoshha M Massoumi R. The Roles of Interleukin-1 Receptor Accessory Protein in Certain Inflammatory Conditions. Immunology. (2022) 166(1):38–46. doi: 10.1111/imm.13462 35231129

[57] Yang Z Gao X Wang J Xu L Zheng Y Xu Y. Interleukin-33 Enhanced the Migration and Invasiveness of Human Lung Cancer Cells. Onco. Targets Ther (2018) 11:843–9. doi: 10.2147/OTT.S155905 PMC5820469 29497316

[58] Tzeng HT Su CC Chang CP Lai WW Su WC Wang YC. Rab37 in Lung Cancer Mediates Exocytosis of Soluble ST2 and Thus Skews Macrophages Toward Tumor-Suppressing Phenotype. Int J Cancer (2018) 143(7):1753–63. doi: 10.1002/ijc.31569 29717487

[59] Chang CP Hu MH Hsiao YP Wang YC. ST2 Signaling in the Tumor Microenvironment. Adv Exp Med Biol (2020) 1240:83–93. doi: 10.1007/978-3-030-38315-2_7 32060890

[60] Qin L Dominguez D Chen S Fan J Long A Zhang M. Exogenous IL-33 Overcomes T Cell Tolerance in Murine Acute Myeloid Leukemia. Oncotarget (2016) 7(38):61069–80. doi: 10.18632/oncotarget.11179 PMC5308636 27517629

[61] Bourgeois E Van LP Samson M Diem S Barra A Roga S. The Pro-Th2 Cytokine IL-33 Directly Interacts With Invariant NKT and NK Cells to Induce IFN-Gamma Production. Eur J Immunol (2009) 39:1046–55. doi: 10.1002/eji.200838575 19266498

[62] Fields JK Kihn K Birkedal GS Klontz EH Sjöström K Günther S. Molecular Basis of Selective Cytokine Signaling Inhibition by Antibodies Targeting a Shared Receptor. Front Immunol (2021) 12:779100. doi: 10.3389/fimmu.2021.779100 35003094 PMC8740070

[63] Sun Z Ji N Ma Q Zhu R Chen Z Wang Z. Epithelial-Mesenchymal Transition in Asthma Airway Remodeling Is Regulated by the IL-33/CD146 Axis. Front Immunol (2020) 11:1598. doi: 10.3389/fimmu.2020.01598 32793232 PMC7387705

[64] Li D Guabiraba R Besnard AG Komai-Koma M Jabir MS Zhang L. IL-33 Promotes ST2-Dependent Lung Fibrosis by the Induction of Alternatively Activated Macrophages and Innate Lymphoid Cells in Mice. J Allergy Clin Immunol (2014) 134:1422–32.e11. doi: 10.1016/j.jaci.2014.05.011 24985397 PMC4258609

[65] John AE Wilson MR Habgood A Porte J Tatler AL Stavrou A. Loss of Epithelial Gq and G11 Signaling Inhibits Tgfβ Production But Promotes IL-33-Mediated Macrophage Polarization and Emphysema. Sci Signal (2016) 9(451):ra104. doi: 10.1126/scisignal.aad5568 27811142

[66] Cayrol C Girard JP. Interleukin-33 (IL-33): A Nuclear Cytokine From the IL-1 Family. Immunol Rev (2018) 281:154–68. doi: 10.1111/imr.12619 29247993

[67] Nie Y Hu Y Yu K Zhang D Shi Y Li Y. Akt1 Regulates Pulmonary Fibrosis *via* Modulating IL-13 Expression in Macrophages. Innate Immun (2019) 25:451–61. doi: 10.1177/1753425919861774 PMC6900639 31299858

[68] Nie Y Sun L Wu Y Yang Y Wang J He H. AKT2 Regulates Pulmonary Inflammation and Fibrosis *via* Modulating Macrophage Activation. J Immunol (2017) 198:4470–80. doi: 10.4049/jimmunol.1601503 28455433

[69] Yi XM Li M Chen YD Shu HB Li S. Reciprocal Regulation of IL-33 Receptor-Mediated Inflammatory Response and Pulmonary Fibrosis by TRAF6 and USP38. Proc Natl Acad Sci USA (2022) 119:e2116279119. doi: 10.1073/pnas.2116279119 35238669 PMC8917384

[70] Akimoto M Hayashi JI Nakae S Saito H Takenaga K. Interleukin-33 Enhances Programmed Oncosis of ST2L-Positive Low-Metastatic Cells in the Tumour Microenvironment of Lung Cancer. Cell Death Dis (2016) 7:e2057. doi: 10.1038/cddis.2015.418 26775708 PMC4816191

[71] Harada H Inoue M Itasaka S Hirota K Morinibu A Shinomiya K. Cancer Cells That Survive Radiation Therapy Acquire HIF-1 Activity and Translocate Towards Tumour Blood Vessels. Nat Commun (2012) 3:783. doi: 10.1038/ncomms1786 22510688 PMC3337987

[72] Babadi AS Kiani A Mortaz E Taghavi K Khosravi A Marjani M. Serum Interleukin-27 Level in Different Clinical Stages of Lung Cancer. Open Access Maced. J Med Sci (2019) 7(1):45–9. doi: 10.3889/oamjms.2019.018 PMC6352477 30740158

[73] Wang CH Chen ZS Bu XM Han Y Shan S Ren T. IL-33 Signaling Fuels Outgrowth and Metastasis of Human Lung Cancer. Biochem Biophys Res Commun (2016) 479:461–8. doi: 10.1016/j.bbrc.2016.09.081 27644880

[74] Jiang Y Liao H Zhang X Cao S Hu X Yang Z. IL-33 Synergistically Promotes the Proliferation of Lung Cancer Cells *In Vitro* by Inducing Antibacterial Peptide LL-37 and Proinflammatory Cytokines in Macrophages. Immunobiology (2020) 225:152025. doi: 10.1016/j.imbio.2020.152025 33190003

[75] Zhou X Feng Y Liu S Li C Teng Y Li X. IL-33 Promotes the Growth of Non-Small Cell Lung Cancer Cells Through Regulating miR-128-3p/CDIP1 Signalling Pathway. Cancer Manag. Res (2021) 13:2379–88. doi: 10.2147/CMAR.S276297 PMC7965692 33737835

[76] Wang K Shan S Yang Z Gu X Wang Y Wang C. IL-33 Blockade Suppresses Tumor Growth of Human Lung Cancer Through Direct and Indirect Pathways in a Preclinical Model. Oncotarget (2017) 8:68571–82. doi: 10.18632/oncotarget.19786 PMC5620278 28978138

[77] Kim BS Clinton J Wang Q Chang SH. Targeting ST2 Expressing Activated Regulatory T Cells in Kras-Mutant Lung Cancer. Oncoimmunology (2020) 9:1682380. doi: 10.1080/2162402X.2019.1682380 32002289 PMC6959450

[78] Schuijs MJ Png S Richard AC Tsyben A Hamm G Stockis J. ILC2-Driven Innate Immune Checkpoint Mechanism Antagonizes NK Cell Antimetastatic Function in the Lung. Nat Immunol (2020) 21:998–1009. doi: 10.1038/s41590-020-0745-y 32747815 PMC7116357

[79] Li X. [The Preliminary Study on the Expression of IL-33/ST2 in Tumor Microenvironment and the Role ofIL-33 on Human CD8(+) T Cells In Vitro]. Soochow University: Master (2015).

[80] Liang Y Wang X Wang H Yang W Yi P Soong L. IL-33 Activates Mtorc1 and Modulates Glycolytic Metabolism in CD8(+) T Cells. Immunology (2022) 165:61–73. doi: 10.1111/imm.13404 34411293 PMC9112898

[81] Gao K Li X Zhang L Bai L Dong W Gao K. Transgenic Expression of IL-33 Activates CD8(+) T Cells and NK Cells and Inhibits Tumor Growth and Metastasis in Mice. Cancer Lett (2013) 335:463–71. doi: 10.1016/j.canlet.2013.03.002 23499895

[82] Qi L Zhang Q Miao Y Kang W Tian Z Xu D. Interleukin-33 Activates and Recruits Natural Killer Cells to Inhibit Pulmonary Metastatic Cancer Development. Int J Cancer (2020) 146:1421–34. doi: 10.1002/ijc.32779 31709531

[83] Zhang J Chen Y Chen K Huang Y Xu X Chen Q. IL-33 Drives the Antitumour Effects of Dendritic Cells *via* Upregulating CYLD Expression in Pulmonary Adenocarcinoma. Artif Cells Nanomed. Biotechnol (2019) 47(1):1335–41. doi: 10.1080/21691401.2019.1596926 30964341

[84] Xu L Zheng Y Wang J Xu Y Xie Y Yang ZP. IL33 Activates Cd8+T and NK Cells Through MyD88 Pathway to Suppress the Lung Cancer Cell Growth in Mice. Biotechnol Lett (2020) 42:1113–21. doi: 10.1007/s10529-020-02815-2 32140881

[85] Feng Y Zhu Y Luo G Wang Z Yu P Zheng L. Expression and Clinical Significance of IL-33 in Patients With non-Small Cell Lung Cancer. Xi Bao Yu Fen Zi Mian Yi Xue Za Zhi (2016) 32:808–11. doi: CNKI:SUN:XBFM.0.2016-06-020 27371849

[86] Chen Y Zhang T Yang Z Chen L Tian J Wang J. Effect of Expression of Interleukin-33, Tumor Microvascular Density and Lymphaticvessel Density on Clinical Prognosis of Patients With Non-Small Cell Lung Cancer. J New Med (2020) 51:370–7. doi: CNKI:SUN:XYXX.0.2020-05-009

[87] Hu LA Fu Y Zhang DN Zhang J. Serum IL-33 as a Diagnostic and Prognostic Marker in Nonsmall Cell Lung Cancer. Asian Pac. J Cancer Prev (2013) 14(4):2563–6. doi: 10.7314/apjcp.2013.14.4.2563 23725175

[88] Yang M Feng Y Yue C Xu B Chen L Jiang J. Lower Expression Level of IL-33 is Associated With Poor Prognosis of Pulmonary Adenocarcinoma. PloS One (2018) 13:e0193428. doi: 10.1371/journal.pone.0193428 29499051 PMC5834175

[89] Taniguchi S Elhance A Van Duzer A Kumar S Leitenberger JJ Oshimori N. Tumor-Initiating Cells Establish an IL-33-TGF-β Niche Signaling Loop to Promote Cancer Progression. Science (2020) 369(6501):eaay1813. doi: 10.1126/science.aay1813 32675345 PMC10870826

[90] Witsch E Sela M Yarden Y. Roles for Growth Factors in Cancer Progression. Physiol (Bethesda) (2010) 25:85–101. doi: 10.1152/physiol.00045.2009 PMC3062054 20430953

[91] Rahman AF Korashy HM Kassem MG. Gefitinib. Prof. Drug Subst. Excip. Relat Methodol. (2014) 39:239–64. doi: 10.1016/B978-0-12-800173-8.00005-2 24794908

[92] Bilsborough J Leung DY Maurer M Howell M Boguniewicz M Yao L. IL-31 is Associated With Cutaneous Lymphocyte Antigen-Positive Skin Homing T Cells in Patients With Atopic Dermatitis. J Allergy Clin Immunol (2006) 117:418–25. doi: 10.1016/j.jaci.2005.10.046 16461143

[93] Robert C Soria JC Spatz A Le Cesne A Malka D Pautier P. Cutaneous Side-Effects of Kinase Inhibitors and Blocking Antibodies. Lancet Oncol (2005) 6:491–500. doi: 10.1016/S1470-2045(05)70243-6 15992698

[94] Dillon SR Sprecher C Hammond A Bilsborough J Rosenfeld-Franklin M Presnell SR. Interleukin 31, a Cytokine Produced by Activated T Cells, Induces Dermatitis in Mice. Nat Immunol (2004) 5:752–60. doi: 10.1038/ni1084 15184896

[95] Wong CK Leung KM Qiu HN Chow JY Choi AO Lam CW. Activation of Eosinophils Interacting With Dermal Fibroblasts by Pruritogenic Cytokine IL-31 and Alarmin IL-33: Implications in Atopic Dermatitis. PloS One (2012) 7:e29815. doi: 10.1371/journal.pone.0029815 22272250 PMC3260155

[96] Gangemi S Franchina T Minciullo PL Profita M Zanghì M David A. IL-33/IL-31 Axis: A New Pathological Mechanisms for EGFR Tyrosine Kinase Inhibitors-Associated Skin Toxicity. J Cell Biochem (2013) 114:2673–6. doi: 10.1002/jcb.24614 23794184

[97] Gewirtz DA. A Critical Evaluation of the Mechanisms of Action Proposed for the Antitumor Effects of the Anthracycline Antibiotics Adriamycin and Daunorubicin. Biochem Pharmacol (1999) 57:727–41. doi: 10.1016/S0006-2952(98)00307-4 10075079

[98] Doroshow JH. Role of Hydrogen Peroxide and Hydroxyl Radical Formation in the Killing of Ehrlich Tumor Cells by Anticancer Quinones. Proc Natl Acad Sci USA (1986) 83:4514–8. doi: 10.1073/pnas.83.12.4514 PMC323764 3086887

[99] Board P. D. Q. A. T. E. Small Cell Lung Cancer Treatment (PDQ®): Patient Version. In: PDQ Cancer Information Summaries. Bethesda (MD: National Cancer Institute (US (2021).

[100] Deng Y Wu S Yang Y Meng M Chen X Chen S. Unique Phenotypes of Heart Resident Type 2 Innate Lymphoid Cells. Front Immunol (2020) 11:802. doi: 10.3389/fimmu.2020.00802 32431711 PMC7214751

[101] Ma Y Wang Q Cao Y Wang G Guo D Abbasi PA. Bio-based and Reduced-Risk Strategies for the Management of Phytophthora Blight and Root Rot of Pepper. In: Arora N. Mehnaz S. Balestrini R. (eds) Bioformulations: for Sustainable Agriculture. Springer, New Delhi. doi: 10.1007/978-81-322-2779-3_9

[102] Harijith A Ebenezer DL Natarajan V. Reactive Oxygen Species at the Crossroads of Inflammasome and Inflammation. Front Physiol (2014) 5:352. doi: 10.3389/fphys.2014.00352 25324778 PMC4179323

[103] Yao Y Chen R Ying C Zhang G Rui T Tao A. Interleukin-33 Attenuates Doxorubicin-Induced Cardiomyocyte Apoptosis Through Suppression of ASK1/JNK Signaling Pathway. Biochem Biophys Res Commun (2017) 493:1288–95. doi: 10.1016/j.bbrc.2017.09.153 28965952

[104] Sanada S Hakuno D Higgins LJ Schreiter ER Mckenzie AN Lee RT. IL-33 and ST2 Comprise a Critical Biomechanically Induced and Cardioprotective Signaling System. J Clin Invest (2007) 117:1538–49. doi: 10.1172/JCI30634 PMC1865027 17492053

[105] Ludke A Akolkar G Ayyappan P Sharma AK Singal PK. Time Course of Changes in Oxidative Stress and Stress-Induced Proteins in Cardiomyocytes Exposed to Doxorubicin and Prevention by Vitamin C. PloS One (2017) 12:e0179452. doi: 10.1371/journal.pone.0179452 28678856 PMC5497966

[106] Sabapathy V Cheru NT Corey R Mohammad S Sharma R. A Novel Hybrid Cytokine IL233 Mediates Regeneration Following Doxorubicin-Induced Nephrotoxic Injury. Sci Rep (2019) 9:3215. doi: 10.1038/s41598-019-39886-9 30824764 PMC6397151

[107] Shani O Vorobyov T Monteran L Lavie D Cohen N Raz Y. Fibroblast-Derived IL33 Facilitates Breast Cancer Metastasis by Modifying the Immune Microenvironment and Driving Type 2 Immunity. Cancer Res (2020) 80:5317–29. doi: 10.1158/0008-5472.CAN-20-2116 PMC7611300 33023944

[108] Guillerey C Stannard K Chen J Krumeich S Miles K Nakamura K. Systemic Administration of IL-33 Induces a Population of Circulating KLRG1(hi) Type 2 Innate Lymphoid Cells and Inhibits Type 1 Innate Immunity Against Multiple Myeloma. Immunol Cell Biol (2021) 99:65–83. doi: 10.1111/imcb.12390 32748462

[109] Lucarini V Ziccheddu G Macchia I La Sorsa V Peschiaroli F Buccione C. IL-33 Restricts Tumor Growth and Inhibits Pulmonary Metastasis in Melanoma-Bearing Mice Through Eosinophils. Oncoimmunology (2017) 6:e1317420. doi: 10.1080/2162402X.2017.1317420 28680750 PMC5486175

[110] Chen X Lu K Timko NJ Weir DM Zhu Z Qin C. IL−33 Notably Inhibits the Growth of Colon Cancer Cells. Oncol Lett (2018) 16(1):769–74. doi: 10.3892/ol.2018.8728 PMC6019937 29963144

[111] Saranchova I Han J Zaman R Arora H Huang H Fenninger F. Type 2 Innate Lymphocytes Actuate Immunity Against Tumours and Limit Cancer Metastasis. Sci Rep (2018) 8:2924. doi: 10.1038/s41598-018-20608-6 29440650 PMC5811448

[112] Moral JA Leung J Rojas LA Ruan J Zhao J Sethna Z. ILC2s Amplify PD-1 Blockade by Activating Tissue-Specific Cancer Immunity. Nature (2020) 579:130–5. doi: 10.1038/s41586-020-2015-4 PMC7060130 32076273

